# Genome wide association study on development and evolution of glutinous rice

**DOI:** 10.1186/s12863-022-01033-1

**Published:** 2022-05-04

**Authors:** Conghui Jiang, Muhammad Abdul Rehman Rashid, Yanhong Zhang, Yan Zhao, Yinghua Pan

**Affiliations:** 1 Rice Research Institute, Guangxi Academy of Agricultural Sciences/Guangxi Key Laboratory of Rice Genetics and Breeding, Nanning, 530007 China; 2 State Key Laboratory of Crop Biology, Shandong Key Laboratory of Crop Biology, College of Agronomy, Shandong Agricultural University, Tai’an, Shandong 271018 PR China; 3 Shandong Rice Engineering Technology Research Center, Shandong Rice Research Institute, Shandong Academy of Agricultural Sciences, Jinan, 250100 China; 4 Department of Bioinformatics and Biotechnology, Government College University, Faisalabad, 38000 Pakistan; 5 State Key Laboratory for Conservation and Utilization of Bio-Resources in Yunnan, Research Center of Perennial Rice Engineering and Technology in Yunnan, School of Agriculture, Yunnan University, Kunming, 650500 China; 6 Institute of Nuclear and Biological Technologies, Xinjiang Academy of Agricultural Sciences, Urumqi, 830091 China

**Keywords:** Glutinous rice, Genome-wide association study, *Oryza Sativa* L., Haplotype analysis, Evolution

## Abstract

**Background:**

Glutinous rice as a special endosperm type is consumed as a staple food in East Asian countries by consumers’ preference. Genetic studies on glutinous rice could be conducive to improve rice quality and understand its development and evolution. Therefor, we sought to explore more genes related to glutinous by genome wide association study and research the formation history for glutinous.

**Results:**

Here, genome-wide association study was performed to explore the associated loci/genes underlying glutinous rice by using 2108 rice accessions. Combining the expression patterns analysis, 127, 81, and 48 candidate genes were identified to be associated with endosperm type in whole rice panel, *indica*, and *japonica* sub-populations. There were 32 genes, including three starch synthesis-related genes *Wx*, *SSG6*, and *OsSSIIa,* detected simultaneously in the whole rice panel and subpopulations, playing important role in determining glutinous rice. The combined haplotype analyses revealed that the waxy haplotypes combination of three genes mainly distributed in Southeast Asia (SEA), SEA islands (SER) and East Asia islands (EAR). Through population structure and genetic differentiation, we suggest that waxy haplotypes of the three genes firstly evolved or were directly inherited from wild rice in *japonica*, and then introgressed into *indica* in SER, SEA and EAR.

**Conclusions:**

The cloning and natural variation analysis of waxy-related genes are of great significance for the genetic improvement of quality breeding and comprehend the history in glutinous rice. This work provides valuable information for further gene discovery and understanding the evolution and formation for glutinous rice in SEA, SER and EAR.

**Supplementary Information:**

The online version contains supplementary material available at 10.1186/s12863-022-01033-1.

## Background

Rice (*Oryza Sativa* L.) is one of the most important crop grain feeding more than half the world’s population [1]. High yield and good quality are two important goals of rice production [2]. Since the green revolution, new farming methods and breeding techniques have greatly increased food production in many countries [3–6]. but the improvement of quality breeding have lagged. The demand of rice with good quality is more urgent for consumers and producers with the improvement of people’s living standards. The yield and quality of rice largely determined by the starch content, the ratio of amylose to amylopectin, and the fine structure of amylopectin.

There are two unique subpopulations of rice, *japonica* and *indica.* But whether in *indica* or *japonica*, it can be divided into glutinous and non-glutinous rice. Rice endosperm type are routinely classified according to their amylose content: high (> 25%), intermediate (20–25%), low (10–19%), very low or soft (3–9%), and waxy or glutinous (< 2%) [7].Compared to non-glutinous rice, the texture of glutinous rice is very sticky. Glutinous rice is a major type of cultivated rice with long-standing cultural importance in Asia, and glutinous rice is also eaten as a staple food of East Asian countries, including Laos and northern Thailand, known as the “center of the glutinous rice region” [8–11]. Therefore, the formation of waxiness in rice is not only affected by natural selection but also human preference.

Starch biosynthesis is a complex system composed of synthesis of substrate adenosine diphosphate glucose, direct starch, and amylopectin [12]. It involves 18 starch synthase enzymes related genes, and each gene plays a different role in various stages of starch synthesis [13–16]. In rice grains, the *Waxy (Wx)* gene encodes granule-bound starch synthase (GBSS), is a major gene controlling amylose synthesis, and directly affects the amylose content (AC). The rice type (glutinous or non-glutinous) is mainly governed by two alleles (*wx* and *Wx)* of same gene [17, 18]. Recently, a study was reported to enrich the range of breeding materials by using a base editing system at the third, fourth, and fifth exon of *Wx*^*b*^ to create a series of mutants with AC of 1.4–11.9% [19]. Rice starch content is a comprehensive trait contributed by a series of starch synthesis genes. A fine regulatory network that regulates the eating and cooking qualities (ECQs) in edible rice has been clarified by association analysis and transgenic verification experiments in the starch biosynthesis pathway [20]. *Wx* and *SSII-3* are two major genes that determine ECQs by affecting AC, gel consistency (GC), and gelatinization temperature (GT). *Wx* is the only gene that has a major effect on AC and GC, and has a minimal effect on GT. Interaction of multiple pairs of genes has a significant effect on rice apparent amylose contents (AAC) [21]. Owing to the interaction among starch-synthesis genes, mutation in a single gene will cause changes in the effects of multiple other genes.

Due to the limitations of traditional parental mapping and the special characteristics of waxiness, only *wx* for waxiness had been cloned and there is molecular evidence of a strong selective sweep in a starch of about ~ 250 kb flanking the *Wx* locus among landraces cultivated in Asian countries [17, 18, 22] More genes related to waxiness are required to be urgently discovered to enrich the natural variation of waxiness and the information about evolutionary origin, domestication and adaptation of key genes of glutinous is conducive to study the formation and evolution of glutinous rice. With the development of sequencing technology, genome wide association study (GWAS) has become an effective mean to discover genes and QTLs for grain qualities [23–25]. The sequencing of 3 K core germplasm rice provides strong guarantee for the discovery of waxy genes [26].

In this study, 2108 rice germplasm were used in GWAS to identify the significant loci and candidate genes controlling the development of glutinous rice. Haplotype analysis was performed to identify the corresponding glutinous haplotypes of three key waxiness related genes. Combined haplotype analyses were carried out to reveal the genetic characteristics of glutinous rice in Southeast Asia (SEA), SEA islands (SER), and East Asia islands (EAR). Phylogenetic tree and population structure analysis for the origin and evolution of three key waxy genes. Our findings provide important information for further gene discovery and, to gain insight into the evolution and formation of glutinous rice in SEA, SER and EAR.

## Results

### Endosperm types analysis within different subgroups

A total of 2108 rice accessions, including 1965 non-glutinous (or non-waxy) and 143 glutinous (or waxy) rice accessions (http://snp-seek.irri.org/), were used to identify waxiness-related genetic loci and analyze the differentiation for development of both endosperm types (Table S1). Meanwhile, 17,132,232 SNPs of the rice panel were obtained from 3KRGP [26]. Subsets of these data were further filtered and used in the subsequent analyses.

**Fig. 1:**
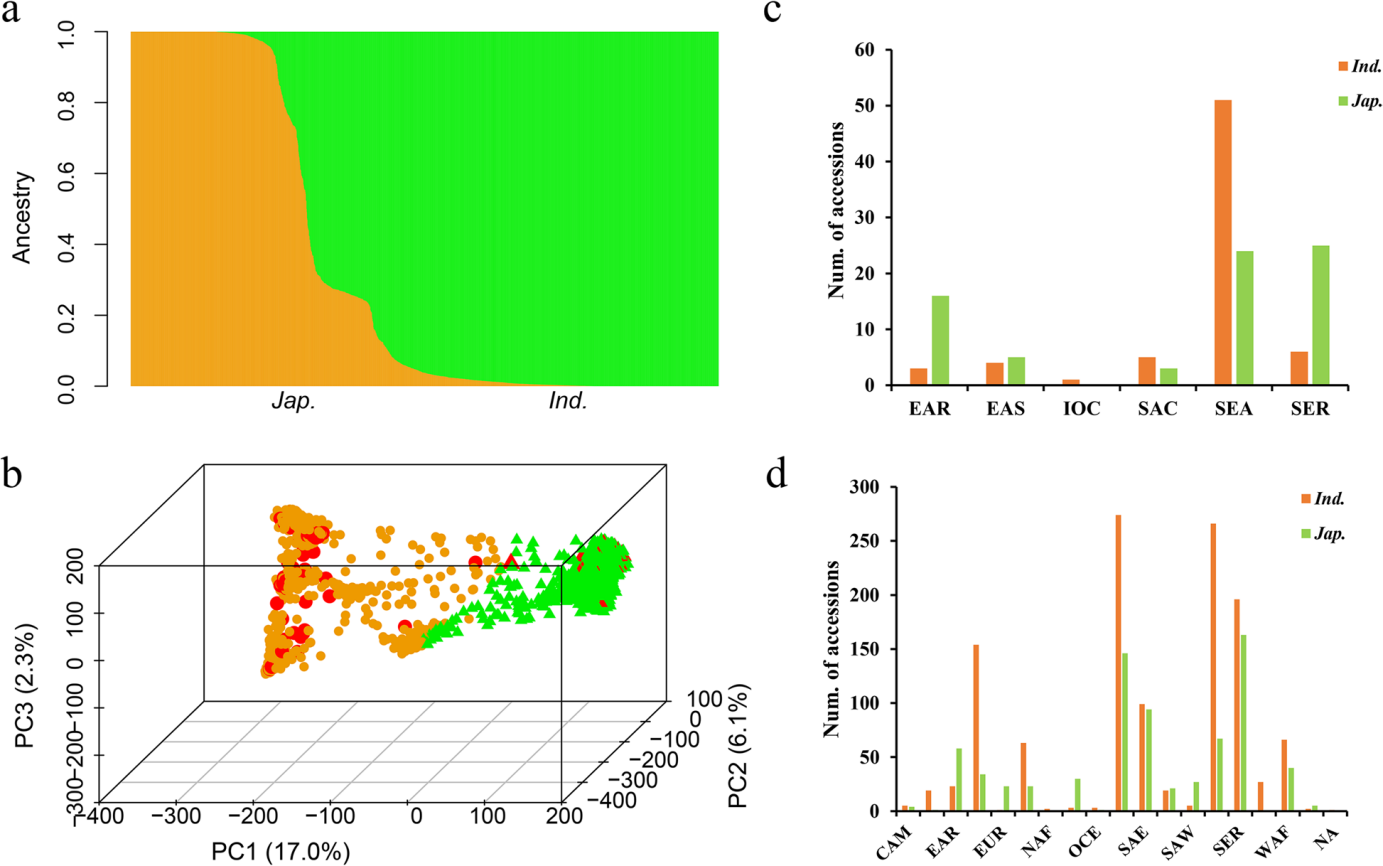
Population structure and geographic distribution of accessions of the association panel. **a** Genetic structure of the panel based on ADMIXTURE for K = 2. **b** Principle components analysis reveals that the first 3 principle components explain 25.4% of the genetic variation within the panel. Green and yellow dot show i indica and japonica, respectively. Red dot indicates glutinous rice accession. **c** and **d** Geographic distribution of glutinous and non-glutinous rice accessions. Geographical information reference to 3010 rice accession (DOI: 10.1038/s41586-018-0063-9), EAR (East Asia Islands), EAS (East Asia), SEA (Southeast Asia), SER (SEA islands), SER-IRRI, OCE (Oceania), SAE (South Asia - East), SAC (South Asia - Central), SAW (South Asia - West), WAS (West Asia), IOC (Indian Ocean), EAF (East Africa), WAF (West Africa), NAF (North Africa), SAM (South America), CAM (Central America and Caribbean), NAM (North America), EUR (Europe), NA(No region information)

Reasonable assessment of population structure is conducive to detect the phenotypic differences and subsequent GWAS of natural population. Using Admixture software [27], we calculated varying levels of K means within the rice population. The *indica* and *japonica* subpopulations appear clearly at *K* = 2 (Fig. 1a). The principal component (PC) analysis indicated that top three PCs explained 17.0, 6.1 and 2.3% of the genetic variation within the rice panel, which supported that there were two main subpopulations (Fig. 1b). Referring to the recent results of 3010 rice accessions [26], we classified the panel into two major subpopulations, 1298 *indica* and 810 *japonica*, although there were several atypical *indica* and *japonica* accessions (Table S1). Hence, the endosperm type of each rice subgroups was compared, and top three PCs were used as covariates to control for subgroup structure in GWAS.

Among the 143 glutinous rice accessions, there were 70 *indica* and 73 *japoncia* (Table S1, Fig. 1b), suggesting broad genetic variation of trait occurred in *indica* and *japonica*. To study the underlying external factors affecting glutinous differentiation, the geographic distribution of accessions with different glutinous traits was investigated. The vast majority of glutinous rice accessions are distributed in SEA, SER and EAR with 75, 31 and 19 accessions, respectively (Fig. 1c). In contrast, non-glutinous rice, as a major endosperm type, was widely distributed in the whole rice growing area (Fig. 1d). This geographic distribution was in consistence with previous research that reported the artificial selection of glutinous rice in Southeast Asia [10, 28]. Taken together, these results suggested that there were large genetic differentiation among glutinous rice accessions, although they were relatively geographically concentrated.

### Identification of waxy trait QTLs by GWAS

**Fig. 2:**
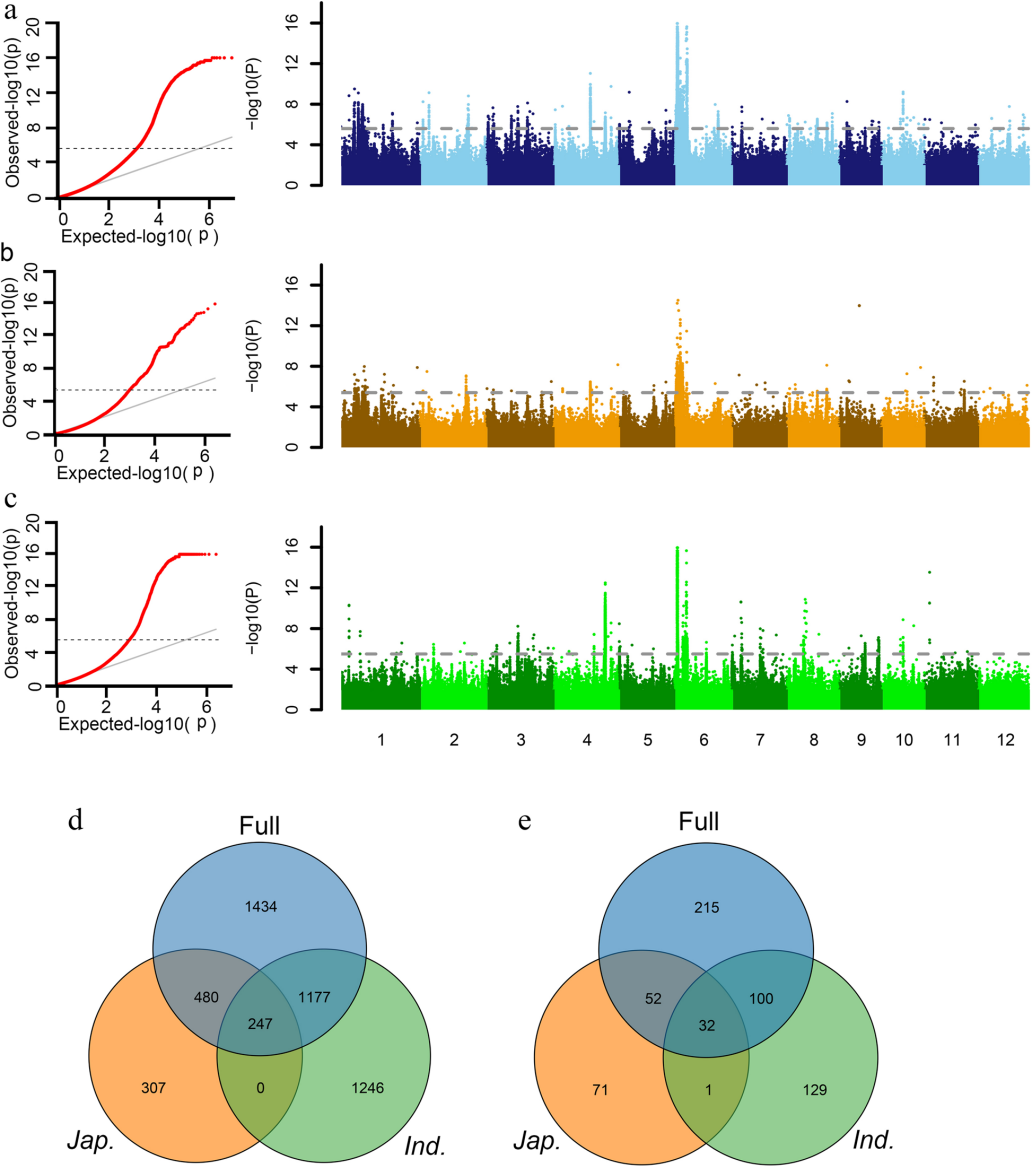
Identification of r waxy trait QTLs by GWAS. Quantile-quantile (Q-Q) plots and Manhattan plots in the whole population (**a**), *japonica* (**b**) and *indica* (**c**). **d** Venn diagram of significant loci detected in different populations. Black four knowngenes identified in this study. **e** Venn diagram of genes with significant loci detected in different populations

Under linear mixed model (LMM) with kinship matrix (K) and top three PCs (Q), GWAS was performed to study the genetic basis of endosperm types. Quantile-quantile (Q-Q) plot showed that LMM efficiently controlled population structure and relationships as there was no inflated *P* values and a majority (95%) of markers exhibited P value equal to or lower than the expected with accordance to null hypothesis (Fig. 2a, b and c). Finally, a total 3338 SNPs located in 399 annotated genes (including gene region and 2 kb promoter region) were identified to associate with endosperm type with threshold of –log(*P*) = 5.6 (Table S2). Taking into account the large genetic differences between the glutinous accessions of *japonica* and *indica* (Fig. 1a and b), we further conducted GWAS of *indica* and *japonica* to explore subpopulation-specific waxy genes. According to the above criteria, a total of 2670 and 1034 associated SNPs were identified in *indica* and *japonica*, located in 262 and 156 annotated genes, respectively (Fig. 2b and c, Table S2). The GWAS detection efficiency of the whole panel was higher with the most associated sites identified (Table S2). By comparing GWAS results of three populations, 1424 significant SNPs (53.3%) of *indica* and 727 significant SNPs (70.3%) of *japonica* could be detected in the whole panel (Table S2). Interestingly, a certain degree of significant loci was detected simultaneously among the whole panel and subpopulations, including 244 common SNPs located in 32 annotated genes (Fig. 2d and e, Table S3), indicating that these genes were important and have conserved gene response for endosperm type between subpopulation.

### Exploration of candidates for endosperm type in rice

**Fig. 3:**
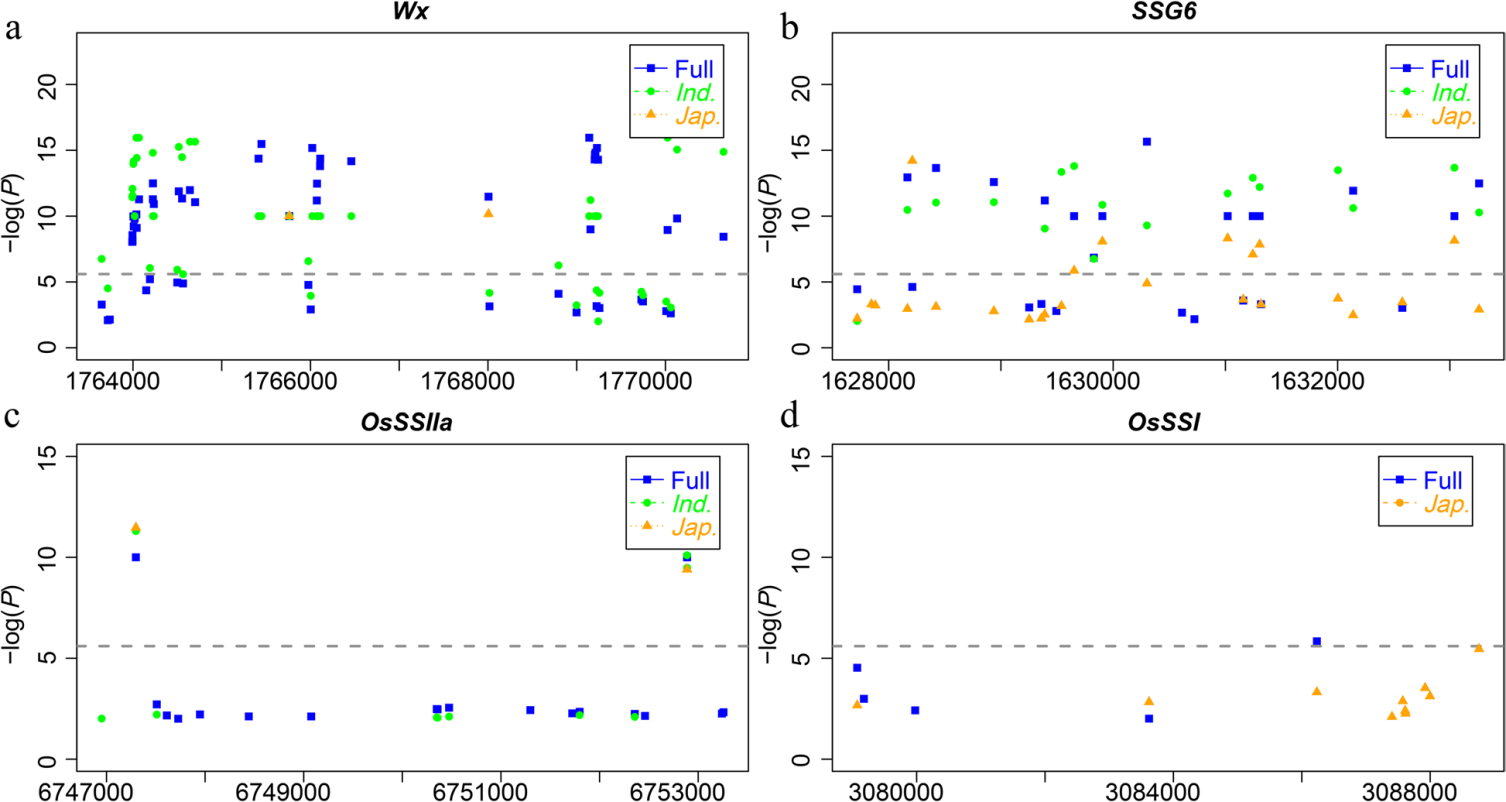
Gene-based association analysis of four starch synthesis-related genes in different population

The real genes related to rice endosperm type were required to be adequately expressed in seeds at grain filling stage such as *OsAGPL2, Wx* and *OsSSIIIa* (Fig. S1). To further screen candidate genes for endosperm types in QTL regions, we firstly analyzed the expression level of candidate genes in rice seeds at two periods (7–8 and 10–14 days after flowering) of seeds development in rice. Among them, 127 of 399 candidates in whole rice panel, 81 of 262 candidates in *indica,* and 48 of 156 candidates in *japonica* showed moderate expression at least one period (FPKM and RPKM > 10), including the 32 annotated genes detected simultaneously in different population (Table S4). To further verify the reliability of combined analysis of GWAS, and expression level, the comparison between GWAS detected candidate genes and the known waxy genes was performed. Three starch synthesis-related genes, *Wx* (*LOC_Os06g04200*), *SSG6* (*LOC_Os06g03990*) and *OsSSIIa* (*LOC_Os06g12450*) were detected among three populations, respectively. As Manhattan plots showed, these known genes showed top signals in whole rice panel and subpopulations (Fig. 3a, b and c). Meanwhile, *OsSSI* (*LOC_Os06g06560*) showed association with endosperm types in the GWAS results of whole rice panel (Fig. 3d). The comparison of the GWAS results with known starch synthesis-related genes indicated that the GWAS results for endosperm type were credible. The four known genes were key loci for natural variations of rice endosperm type, and the other 28 genes are important gene for endosperm type needed to be further verified by transgenic experiment.

### Natural variation in three key genes responsible for rice endosperm type

**Fig. 4:**
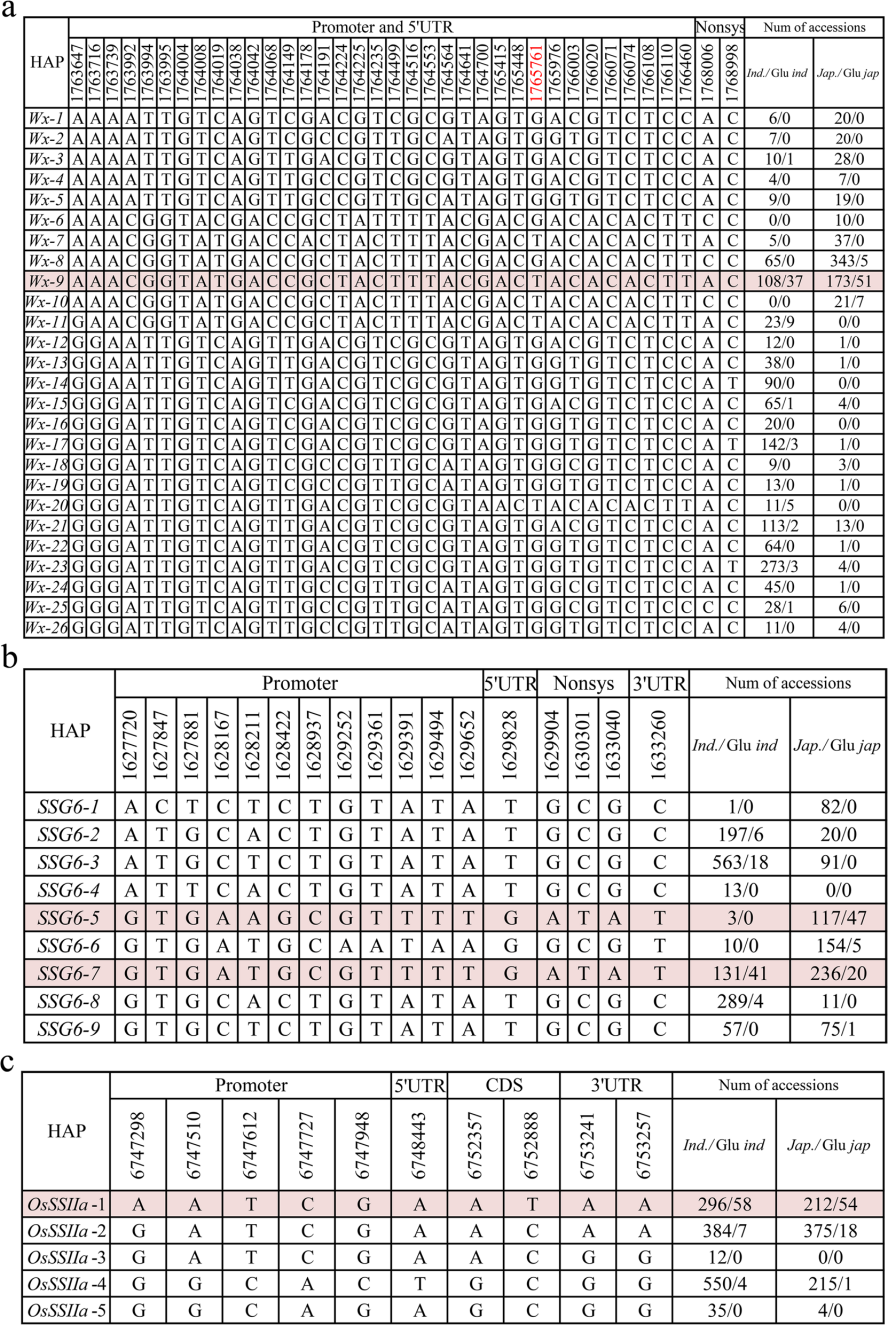
Haplotype analyses for three key cloned genes conferring waxy traits. Haplotypes filled in color are waxy haplotypes. Number on the left and right represent the number of total accessions and glutinous accessions in *indica* and *jaoponica*, respectively

The exploration of natural variation of key endosperm type is beneficial in breeding for high-quality rice. We performed haplotype analysis to identify their elite alleles of the three key genes (*Wx*, *SSG6* and *OsSSIIa*) for rice endosperm types. Firstly, association analysis of candidate genes was performed between endosperm types and 537 SNPs with MAF > 0.01 located in three known genes. Of these, 100 SNPs were significant associated with rice endosperm type (−log(*p*) > 2). Here, we focused on non-synonymous SNPs, SNPs at splice site and SNPs in promoter (Table S5), as these SNPs could be responsible for functional variation through changes in expression and protein sequence [29–31]. A total of 37 significant SNPs were identified within *Wx* gene, including two non-synonymous SNP, one SNP at split site, and 34 in promote or UTR regions. Twenty-six haplotypes, named *Wx-1* to *Wx-26*, were identified in whole panel (Fig. 4a). Twenty-four of 26 haplotypes were detected in *indica*, eight of which showed moderate frequencies ranging from 5 to 23.3%. By comparison, 47.8 and 24.1% *japonica* carried *Wx-8* and *Wx-9*, suggesting there were large genetic variation of *Wx* in *indica* than *japonica* (Fig. 4a). Previous studies showed that Chr6_1765761 was a key functional SNP for post-transcriptional modification of *Wx* [32] The mutant of fifth exons of *Wx*^*b*^ induced to lower AC than that of glutinous rice. In our study, we did not detect a unique waxy haplotype of *W*x. *Wx-9* (allele T at Chr6_1765761) considered as the waxy haplotype, with 37 of 108 in *indica* and 51 of 173 in *japonica* were glutinous rice (Fig. 4a and Fig. S2). The results suggested that *Wx* was not the only key gene accounted for natural variation in proportion of amylopectin and amylose in rice. Waxiness of rice, as a physiological trait, is often the result of the continuous joint change of multiple biochemical processes of starch biosynthesis.

Based on 17 significant SNPs within *SSG6* (twelve in the promoter, one in the 5’UTR, 3 non-synonymous SNPs and one in the 3’UTR), nine major haplotypes, named *SSG6*–1 to *SSG6*–9, were identified in whole panel. *SSG6*–2, *SSG6*–3, *SSG6*–7 and *SSG6*–8 were predominantly represented *indica* varieties, accounting for 15.2, 43.4, 10.1 and 22.3% of the total, respectively. Moreover, *SSG6*–1, *SSG6*–3, *SSG6*–5, *SSG6*–6 and *SSG*6–7 were predominant within *japonica*, accounting for 10.1, 11.2, 14.4, 19.0 and 29.1% (Fig. 4b). The results indicated the existence of a certain degree of genetic differentiation of *SSG6* between *indica* and *japonica,* although there were two shared haplotypes between *indica* and *japonica*. Further study showed that *SSG6*–7 could be considered as main waxy haplotype, due to that 41 of 131 *indica* and 20 of 236 *japonica* carrying *SSG6*–7 were glutinous rice. Additionally, a *japonica*-special glutinous haplotype *SSG6*–5 was detected with 47 of 117 *japonica* carrying *SSG6*–5 were glutinous rice (Fig. 4b and Fig. S2). Meanwhile, we detected 5 haplotypes (named *OsSSIIa*-1 to *OsSSIIa*-5) of *OsSSIIa* gene, based on 10 significant SNPs (five in promoter, one in 5’UTR, two non-synonymous SNPs and two in 3’UTR). *OsSSIIa*-1, *OsSSIIa*-2 and *OsSSIIa*-3 were predominant in whole panel (Fig. 4c). There was no obvious genetic differentiation of *OsSSIIa* between *indica* and *japonica*. *OsSSIIa*-1 could be considered as waxy haplotype, as 58 of 296 *indica* and 54 of 212 *japonica* accessions carrying *OsssIIa*-1 were glutinous rice (Fig. 4c and Fig. S2).

**Fig. 5:**
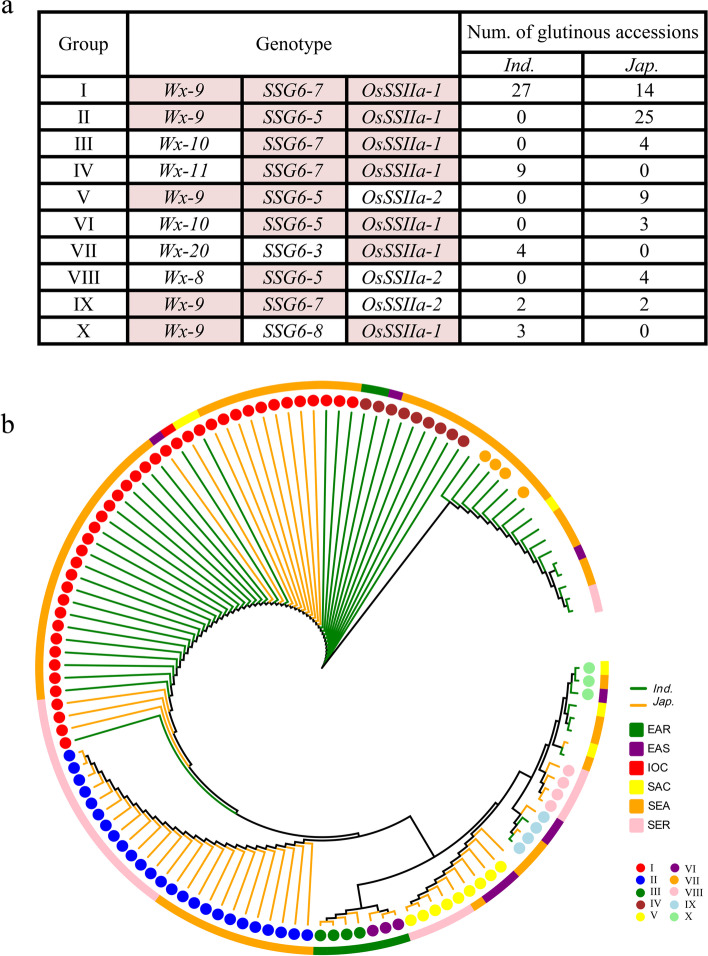
Combined haplotypes and phylogenetic tree of haplotype combinations based on the haplotype of *Wx*, *SSG6* and *OsSSIIa*. **a** Combined haplotypes of *Wx*, *SSG6* and *OsSSIIa* in 124 glutinous accessions. The haplotype combinations with more than 3 accessions are listed. Haplotypes filled in color are waxy haplotypes. **b** The phylogenetic tree based on combined haplotypes of *Wx*, *SSG6* and *OsSSIIa* in 124 glutinous accessions. Linear represent subpopulation. Colored squares represent different regions. Colored circles represent different combined haplotypes

Taken together, we identified the key glutinous rice haplotype of each gene (Fig. S2), although none of them completely determined the waxiness of rice. Furthermore, it provides an important message that waxiness of rice, as a physiological trait, is also determined by a complex network, rather than simple genes in the biochemical synthesis pathway in the traditional sense. To prove the above hypothesis, we first examined the geographical distribution of different haplotype combinations of the three genes. Totally, there were 27 haplotype combinations in 124 glutinous rice accessions, haplotype combinations with more than three accessions were listed (Fig. 5a). Among 75 glutinous rice of SEA, 33 accessions carried the haplotype combination of *Wx*-9, *SSG6*–7 and *OsSSIIa*-1, 12 accessions carried the haplotype combination of *Wx*-9, *SSG6*–5 and *OsSSIIa*-1. The haplotype combination of *Wx*-9, *SSG*6–5 and *OsSSIIa*-1 was also the predominant in SER, while most glutinous accession of EAR carried the haplotype combinations of *Wx*-10, *SSG6*–7 and *OsSSIIa*-1 or *Wx*-10, *SSG6*–5 and *OsSSIIa*-1 (Fig. 5b), indicating the combination of the waxy haplotypes of three genes were conductive to the formation of glutinous rice in SEA, SER and EAR.

### Population structure and genetic differentiation of three key genes between both endosperm types

**Fig. 6:**
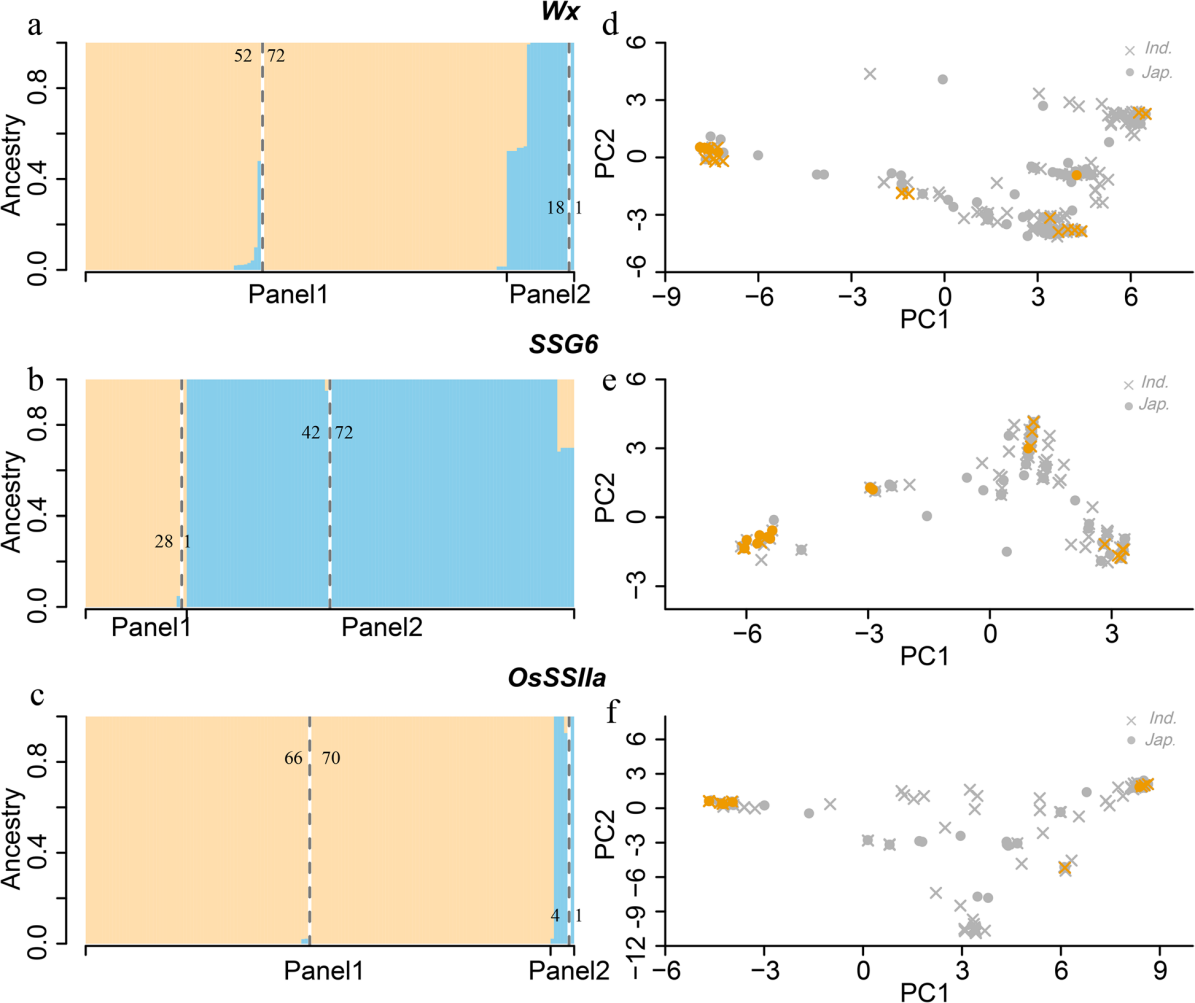
Admixture patterns and principal component analysis for three key genes for waxy traits based on SNPs within gene region. Navajowhite and skyblue represent Panel1 and Panel2 analyzed by the software Admixture. Here only 143 glutinous rice were shown. The numbers on the left and right of Panel1 or Panel2 represent the number of glutinous *indica* and *japonica*,respectively. For PC analysis, grey and orange represent non-glutinous and glutinous rice

The sequence alignment of three key waxy genes and geographical distribution of their different haplotype combinations suggested that the genetic differences underling waxiness trait among regions was greater than that between subpopulations in rice. To confirm the above hypothesis, we investigated the population structure and admixture patterns of each gene in the whole rice panel. We first estimated ancestry proportions of *Wx*, *SSG6* and *OsSSIIa* for individuals by Admixture. Population structure based on each of three genes showed different genetic structures from the whole genome. Admixture model using 202 SNPs within *Wx* gene indicated that 53 of 70 glutinous *indica* accessions clustered with glutinous *japonica* accessions (Fig. 6a). Meanwhile, admixture model using 123 SNPs within SSG6 gene indicated that 42 of 70 glutinous *indica* accessions clustered with glutinous *japonica* accessions, and one glutinous *japonica* accession clustered with other 28 glutinous *indica* accessions (Fig. 6b). Additionally, admixture model using 194 SNPs within *OsSSIIa* gene showed that 66 of 70 glutinous *indica* accessions clustered with glutinous *japonica* accessions, and one glutinous *japonica* accession clustered with other 4 glutinous *indica* accessions (Fig. 6c). The results confirmed that there was no obvious genetic differentiation of the three key waxy genes between *japonica* and *indica* distributed in SEA, SER and EAR, which was supported by further PC analysis (Fig. 6d, e and f).

**Fig. 7:**
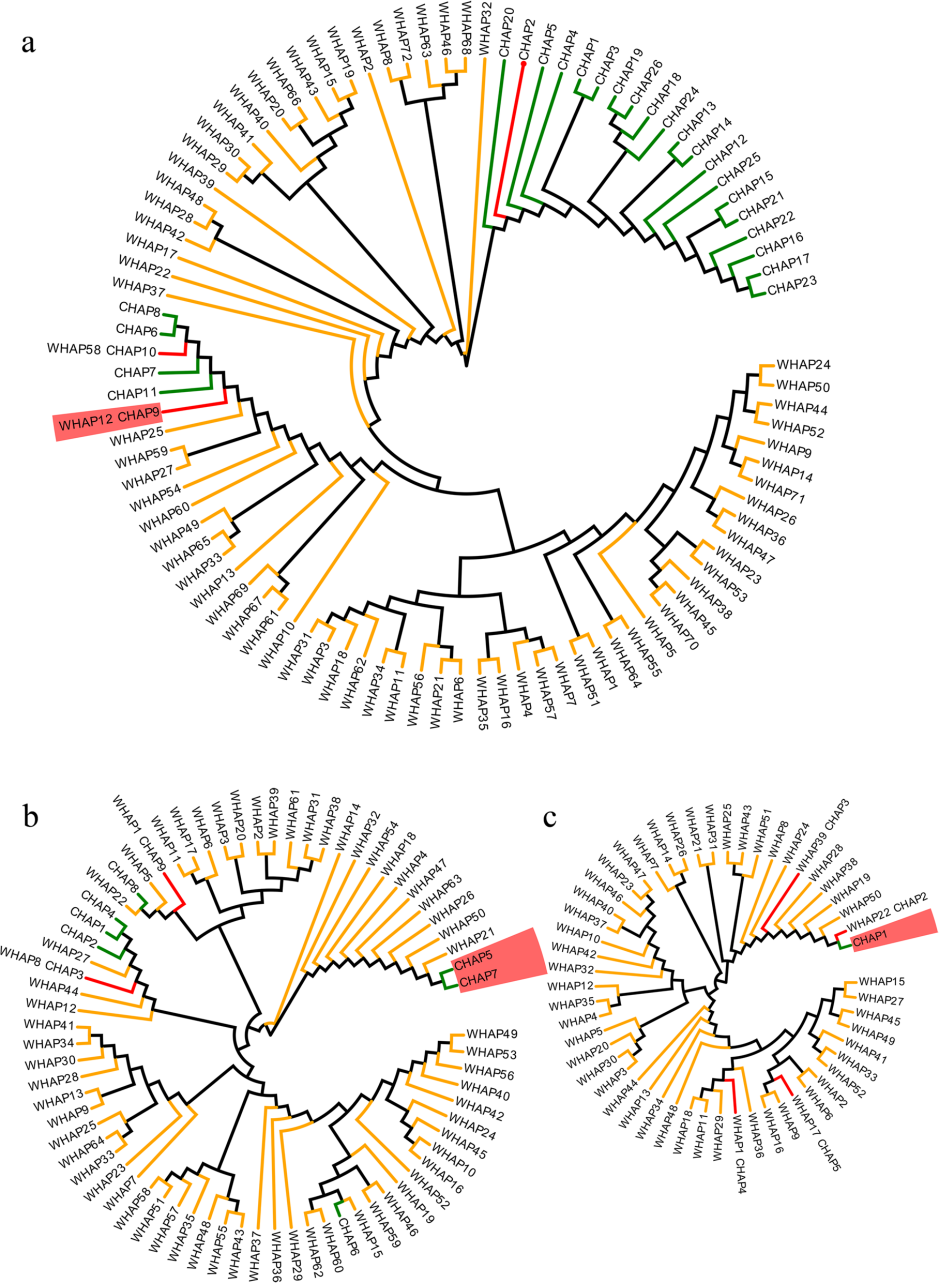
Phylogenetic tree of three keys gene based on haplotypes on cultivated rice and wild rice. **a**, **b** and **c** represent *Wx*, *SSG6* and *OsSSIIa*. The prefix W stands for wild rice and the prefix C stands for cultivated rice. Green, orange and red linear represent haplotypes in cultivated, wild rice and shared between them, taxa name with red background represents waxy haplotypes of each gene

The exceptional genetic similarity among glutinous rice revealed by PC and admixture analyses could be caused by a unique domestication process. The origin of waxy haplotypes of the three genes and how they spread in *japonica* and *indica* rice are two key issues to reveal the formation of glutinous rice. Here, we firstly examined haplotypes of three known genes in wild rice. There were 72, 64 and 52 haplotypes in *Wx*, *SSG6* and *SSIIa* of wild rice. The waxy haplotypes *Wx-9* of Wx gene could be detected in 3 wild rice accessions, which were from Thailand and China. The results indicated that the waxy haplotype *Wx-9* could be inherited from wild rice, but it is a very unlikely scenario that all waxy haplotype in both rice subpopulations originate directly from a small amount of wild rice (Fig. 7a). Additionally, none of wild rice carried waxy haplotypes *SSG6–5* and *SSG6–7* of *SSG6* and waxy haplotype *OsSSIIa-1* of *OsSSIIa* (Fig. 7b and c), suggesting that the waxy haplotypes of *SSG6* and *OsSSIIa* newly generated during rice domestication. Taken together, a more possible hypothesis for the exceptional genetic similarity among glutinous rice is substantial local gene flow of *Wx*, *SSG6* and *SSIIa* between *indica* and *japonica* in SEA, EAR, and EAR.

**Fig. 8:**
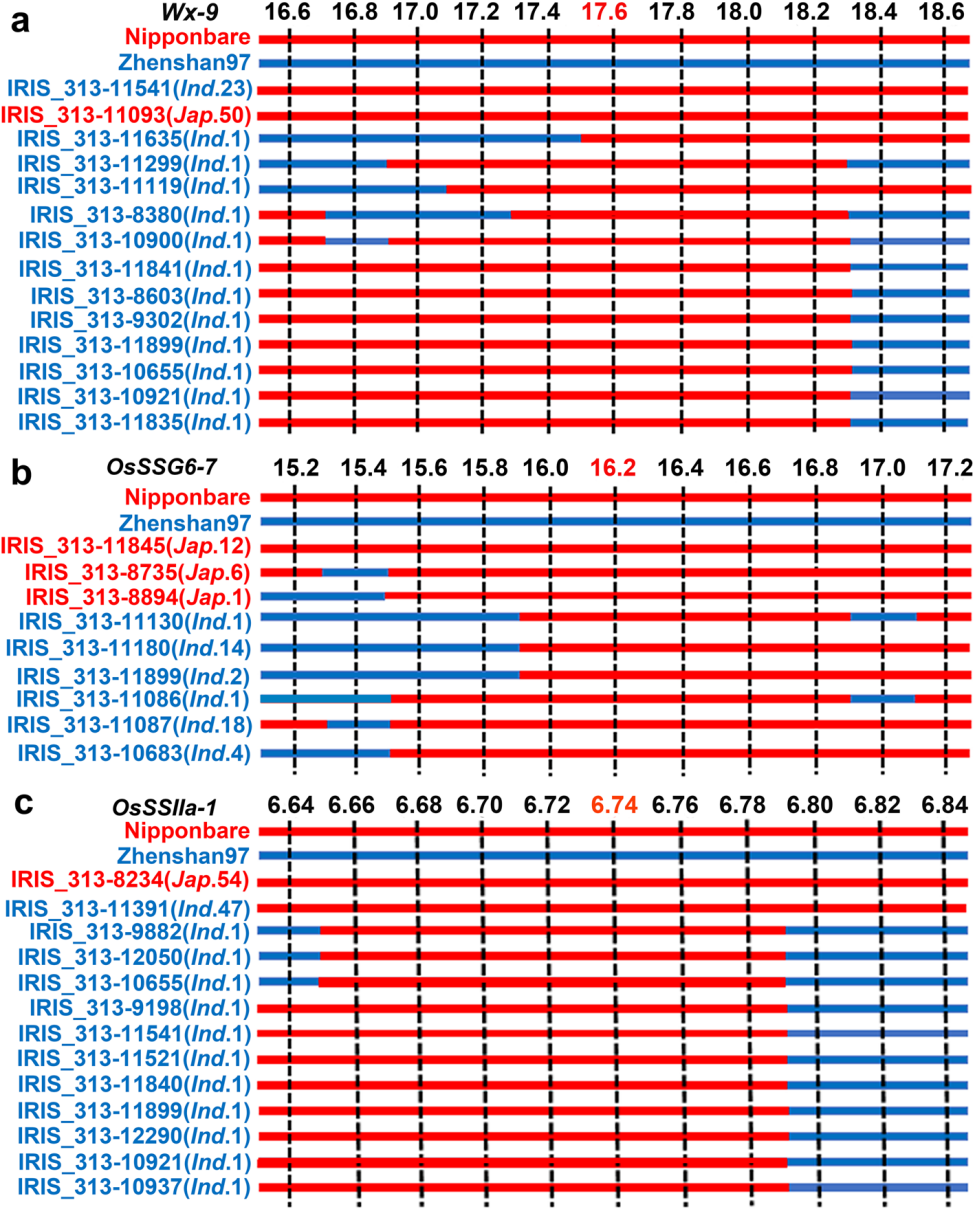
Analyses of introgressed regions for *Wx, OsSSG6* and *OsSSII.* Eleven SNP markers in 200 kb upstream and downstream intervals of these genes were used. *Ind., indica*. *Jap. japonic*a. Nipponbare and Zhenshan97 were reference sequences. Red and blue bars represent *japonica* and *indica* genotypes respectively. Markers at 17.6, 16.2 and 6.74 Mb were the closest to *Wx*, *OsSSG6* and *OsSSIIa* respectively. The number next to *Ind*. and *Jap.* represent the number of glutinous *indica* and *japonic*a, respectively

To further determine the hypothesis of gene flow and examine the direction of gene flow, we performed phylogenetic analyses using all haplotype types of each gene. For *Wx* gene, waxy haplotype *Wx-9* clustered with other *japonica* haplotypes and formed a monophyletic group (Fig. 7a). Meanwhile, two waxy haplotypes *SSG6–5* and *SSG6–7* of SSG6 clustered together with long genetic distance to other haplotypes of cultivated rice (Fig. 7b). Additionally, waxy haplotype *SSIIa-1* clustered with *SSIIa-2* and two wild haplotypes (Fig. 7c). Phylogenetic trees in cultivated rice indicated that the waxy haplotypes of each gene were closer to their corresponding *japonica* haplotypes than *indica* haplotypes, such as *Wx-9* closed to *Wx-7/8/10*, S*SG6–5/7* closed to *SSG6–6*, and *OsSSIIa-1* mainly closed to *japonica* as the haplotype *OsSSIIa-2* account for 46.5% of total in *japonica* accessions (Fig. S3). Further analyses of introgressed regions in 143 glutinous rice showed that the waxy haplotype *Wx-9, SSG6–7 and OsSSIIa-1* were transferred from *japonica* to the *indica* population (Fig. 8). Above all, we suggested that glutinous haplotypes of the three genes in *japonica* rice firstly evolved or were directly inherited from wild rice, and then introgressed into *indica* rice in SER, SEA and EAR.

## Discussion

### Waxiness is a complex polygenic trait

The endosperm type is an important characteristic of rice quality. Glutinous rice is one of the traditional grains loved by mankind. There are various reports of research on waxiness in the past decades, but only *Wx* was cloned. In order to analyze the genetic composition more accurately, it is necessary to identify more genes that regulate this trait. Through genome wide association analysis in this study, a large number of waxiness-related loci were identified, indicating that the waxiness trait is a complex quantitative trait controlled by multiple genes. The *Wx*, *SSG6* and *OsSSIIa* as the starch synthesis controlling genes were identified in full population and subpopulations with strong correlation signals in GWAS result, are proved as key genes controlling waxiness. Sequence analysis showed several alleles of *Wx* for AC [7, 32–36]. Previous studies showed that the base substitutions in coding sequence of *ALK* may cause the alteration in gelatinization temperature and the mutation in *ssg6* introduced a premature stop codon developed enlarged starch grains in endosperm [37–39]. In this study, natural variations for waxiness in three key genes were investigated on the basis of 2108 rice accessions. The 26, 9, and 5 haplotypes of *Wx*, *SSG6*, and *OsSSIIa* were found, respectively. Further analysis showed that *Wx-9*, *SSG6–5/7* and *OsSSIIa-1* were considered as the waxy haplotypes. The research provides important breeding variations for glutinous rice breeding.

### Three key genes cooperate in evolution of glutinous rice

In previous studies, *Wx* was observed as the key gene for waxiness. Other widely conducted evolutionary studies and the manipulation of *Wx* in rice breeding indicated that the *Wx* cooperated with others starch synthesis gene to form a fine regulating network that controls the eating and cooking quality [7, 20, 35, 40]. In our research, through the combination of haplotypes, the interaction between the three genes (*Wx*, *SSG6*, *OsSSIIa*) was studied, which provided the reference for molecular pyramid breeding. The combined haplotype analysis of *Wx, SSG6* and *OsSSIIa* showed *Wx-9 / SSG6–5(7)* and *OsSSIIa-1* genotypes may be the best allele combination for waxy rice and quality breeding, because the combination of these three alleles accounted for the largest proportion in the main distribution area of waxy materials. This study revealed the potential gene combination types of glutinous rice that are popular in Southeast Asia. Population structure and genetic differentiation of three waxiness-related genes showed that glutinous haplotypes of the three genes in *japonica* rice firstly generated or were directly inherited from wild rice, and then flowed into *indica* rice in SER, SEA and EAR. Although the mechanism of these genes to control waxiness is still not clear, elucidating their molecular characteristics and evolutionary patterns in rice germplasm will help to promote glutinous rice breeding. These cloned genes and ongoing gene cloning work will provide a comprehensive understanding of mechanism behind the waxiness, which can then be applied to design varieties of the desired quality.

## Conclusions

As a globally known staple food, rice is well domesticated in the world. Various regions have different food preferences, which lead to design the goals for breeding programs. Glutinous rice as a special endosperm type is also consumed as a staple food in East Asian countries by consumers’ preference. But no genetic study on development and evolution of glutinous rice, specifically in this region has been reported. Here, 2108 rice germplasm were used in GWAS to identify the significant loci and candidate genes controlling the development of glutinous rice. Candidate genes were screened in whole rice panel, *indica*, and *japonica* sub-populations with transcriptome analysis. There were 32 genes, including three starch synthesis-related genes *Wx*, *SSG6*, and *OsSSIIa*, detected simultaneously in the whole rice panel and subpopulations, playing important role in determining glutinous rice. Combined haplotype analysis revealed that the waxy combined haplotype of three genes mainly distributed in Southeast Asia (SEA), SEA islands (SER) and East Asia islands (EAR). This study provides valuable information for further gene discovery and understanding the evolution and formation for glutinous rice in SEA, SER and EAR.

## Methods

### Plant material

A total of 2108 cultivated rice varieties were used in the present study, which were obtained from the 3000 Rice Genome Project (3KRGP) [26, 41]. The phenotype of endosperm type for the 3000 rice varieties collected from the International Rice Genebank Collection Information System (IRGCIS) was recorded in phenotype data dictionary. The endosperm type recorded as three grades, non-glutinous (non waxy) glutinous (waxy) and intermediate. In our study, 1965 non-glutinous (or non-waxy) and 143 glutinous (or waxy) rice were used to study glutinous (http://snp-seek.irri.org/). Additionally, 446 wild rice accessions from a previous report were used to study evolutionary aspects [28].

### Population structure and genetic differentiation

In the whole accessions panel, 5,039,852 independent SNPs across the whole genome determined by PLINK (window size 50, step size 50, *R*^2^ ≥ 0.3) [42] were used to population structure and admixture patterns analysis by Admixture and GAPIT [43, 44]. For the population structure against the three known genes, SNPs located in gene region and 2 kb promoter region were used. Meanwhile, these SNPs were used to construct neighbor-joining tree in accessions panel with and/or without 446 wild rice accessions. Neighbor-joining tree were developed in MEGA version 7 with the bootstrap method and 1000 replicates [45].

### Genome-wide association study

Total of 5,039,852 SNPs with missing rates ≤50% and minor allele frequencies ≥5% obtained from 3000 Rice Genome Project (3KRGP) using an in-house Perl script. GWAS were performed using GAPIT under the LMM model [46, 47]. Here, the top three principal components (PCs) were used to estimate population structure. Given that it was too stringent for significant association detection when the threshold was derived from the total number of markers [47], the threshold to control the type I error rate was defined at –log(*p*) = 5.6 after Bonferroni-adjusted correction [48].

### Candidate genes expression analysis

According to the results of the association analysis, genes with significant loci were screened, including gene region and 2 kb promoter region. To study the expression pattern of each gene, two sets of transcriptome data of rice seeds at two periods (GSE98924 for 7–8 days and GSE132303 for 10–14 days after flowering) were obtained from NCBI (https://www.ncbi.nlm.nih.gov/guide/genes-expression), Gene expression with the value of FPKM or RPKM > 10 in at least one set deemed as stable expression gene, then genes with stable expression in rice seeds were selected as candidate genes.

### Haplotype analysis

Based on information on coding sequence (CDS) coordinates and the transcript from MSU RGAP 7, we separated non-synonymous SNPs, SNPs at splice site and SNPs in promoter from all SNPs across the 2108 accessions using an in-house Perl script. Non-synonymous SNPs, SNPs at splice site and SNPs in promoter significant associated with rice endosperm type (−log(*p*) > 2) were used for haplotype analysis.

### Introgressed regions analyses

Use the genotypes of the 3 K database to perform introgressed analysis, mainly for materials containing waxy haplotypes with Wx-9 for *Wx*, OsSSIIa-1 for *OsSSIIa* and OsSSG6–7 for *OsSSG6* in 143 glutinous. Eleven SNP markers evenly distributed in 200 kb upstream and downstream intervals of these genes were used. Nipponbare and Zhenshan97 with better sequencing quality were the reference sequences as *japonica* and *indica*, respectively.

## Supplementary Information

**Additional file 1: Supplementary Table S1**: List and brief introduction of 2108 rice accessions used in this study.

**Additional file 2: Supplementary Table S2**: Summary of SNPs associated significantly with glutinous detected in full population, indica and japonica by GWAS mapping.

**Additional file 3: Supplementary Table S3**: Thirty two candidate genes ovelapped in full population, indica, and japonica

**Additional file 4: Supplementary Table S4**: Expression analysis of candidate genes in full population, indica and japonica.

**Additional file 5: Supplementary Table S5**: SNPs used for haplotype analysis in three key genes.

**Additional file 6: Figure S1**. Expression patterns of *OsAGPL2*, *Wx* and *OsSSIIIa* by Rice eFPBrowser (http://bar.utoronto.ca/efprice/cgi-bin/efpWeb.cgi). **Figure S2**. The proportion of glutinous rice among different haplotypes of three key genes. **Figure S3**. Phylogenetic tree based on haplotypes of three keys genes in cultivated rice.

## Data Availability

The endosperm type of 2108 cultivated rice varieties used in our study can be obtained from the 3 K-RG dataset: http://snp-seek.irri.org. The 3 K-RG sequencing data used for our analyses can be obtained via project accession PRJEB6180 from NCBI (https://www.ncbi.nlm.nih.gov/sra/?term= PRJEB6180). The transcriptome data of rice seeds at 7-8 days and 10-14 days after flowering were obtained from NCBI (https://www.ncbi.nlm.nih.gov/gds), the series accession ID: GSE98924 and GSE132303.

## Abbreviations

GWAS: Genome-wide association study
AC: Amylose content
ECQs: Eating and cooking qualities
GC: Gel consistency
GT: Gelatinization temperature
AAC: Apparent amylose contents
EAR: East Asia Islands
EAS: East Asia
SEA: Southeast Asia
SER: SEA islands
IRRI: Philippines
OCE: Oceania
SAE: South Asia - East
SAC: South Asia - Central
SAW: South Asia - West
WAS: West Asia
IOC: Indian Ocean
EAF: East Africa
WAF: West Africa
NAF: North Africa
SAM: South America
CAM: Central America and Caribbean
NAM: North America
EUR: Europe
NA: No region information
