# Supplementary material for: Genome wide association study on development and evolution of glutinous rice
Source: BMC Genom Data. 2022 May 4;23:33. doi: 10.1186/s12863-022-01033-1 (PMC9066796; doi:10.1186/s12863-022-01033-1)
Supplement: Supplementary file 6 — Additional file 6: Figure S1. Expression patterns of OsAGPL2, Wx and OsSSIIIa by Rice eFPBrowser (http://bar.utoronto.ca/efprice/cgi-bin/efpWeb.cgi). Figure S2. The proportion of glutinous rice among different haplotypes of three key genes. Figure S3. Phylogenetic tree based on haplotypes of three keys genes in cultivated rice. [file 12863_2022_1033_MOESM6_ESM.docx]

**Genome wide association study on development and evolution of glutinous rice**

Conghui Jiang^3^, Muhammad Abdul Rehman Rashid^4,5^, Yanhong Zhang^6^, Yan Zhao^2*^, Yinghua Pan^1*^

^1^ Rice Research Institute, Guangxi Academy of Agricultural Sciences/Guangxi Key Laboratory of Rice Genetics and Breeding, Nanning, 530007, China

^2^ State Key Laboratory of Crop Biology, Shandong Key Laboratory of Crop Biology, College of Agronomy, Shandong Agricultural University, Tai'an, Shandong, 271018, PR China

^3^ Shandong Rice Engineering Technology Research Center, Shandong Rice Research Institute, Shandong Academy of Agricultural Sciences, Jinan 250100, China

^4^ Department of Bioinformatics and Biotechnology, Government College University, Faisalabad, 38000, Pakistan

^5^ State Key Laboratory for Conservation and Utilization of Bio-Resources in Yunnan, Research Center of Perennial Rice Engineering and Technology in Yunnan, School of Agriculture, Yunnan University, Kunming 650500, China

^6^ Institute of Nuclear and Biological Technologies, Xinjiang Academy of Agricultural Sciences, Urumqi, 830091, China

^*^Corresponding Authors: Yinghua Pan ([panyinghua2008@163.com](mailto:panyinghua2008@163.com), Tel: +86-18077779296) and Yan Zhao ([zhaoyan1216@163.com](mailto:zhaoyan1216@163.com), [Tel: +86-13011202855](Tel:13011202855))


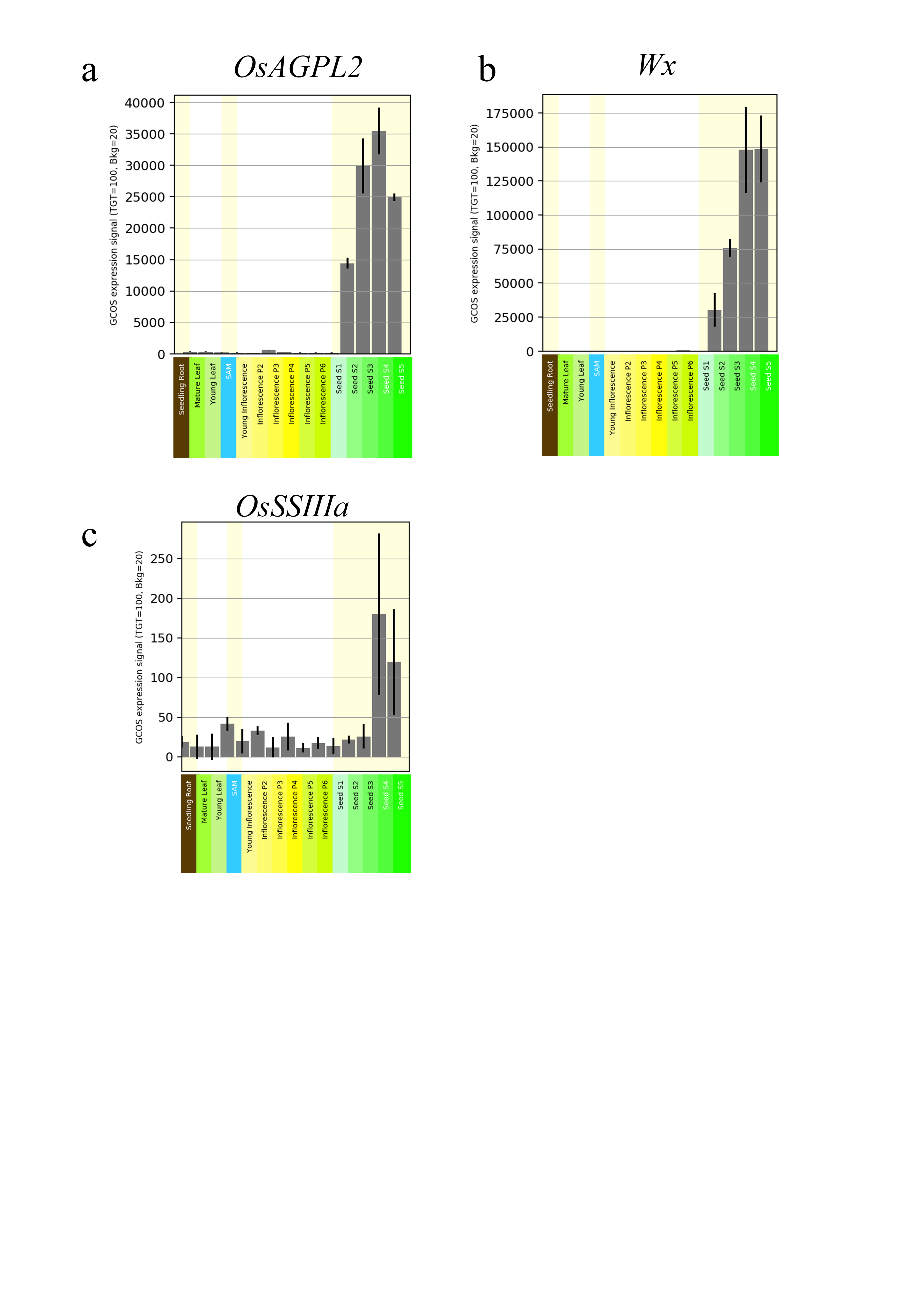


Fig.S1. Expression patterns of *OsAGPL2*, *Wx* and *OsSSIIIa* by Rice eFPBrowser (http://bar.utoronto.ca/efprice/cgi-bin/efpWeb.cgi).


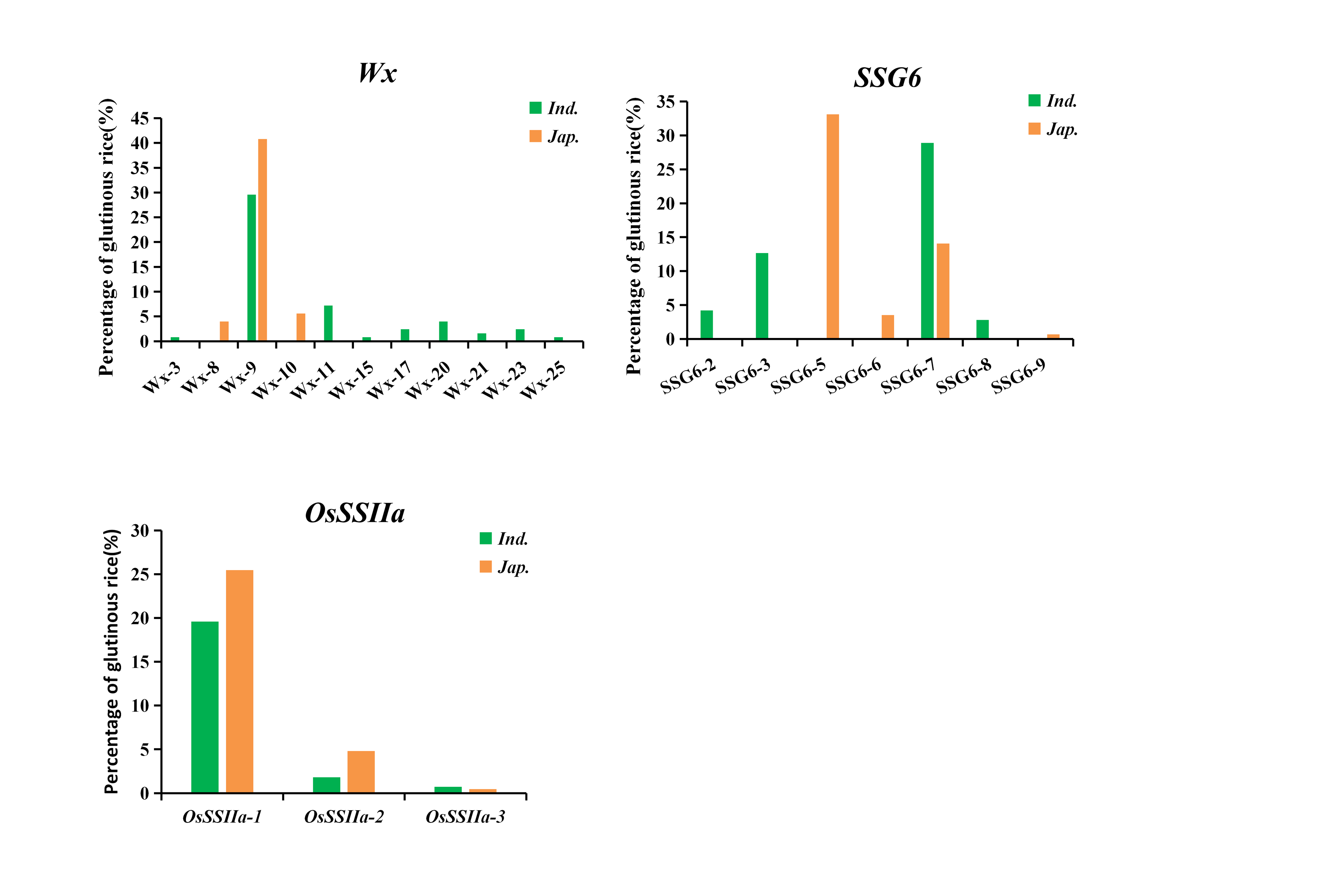


Fig.S2 The proportion of glutinous rice among different haplotypes of three key genes.


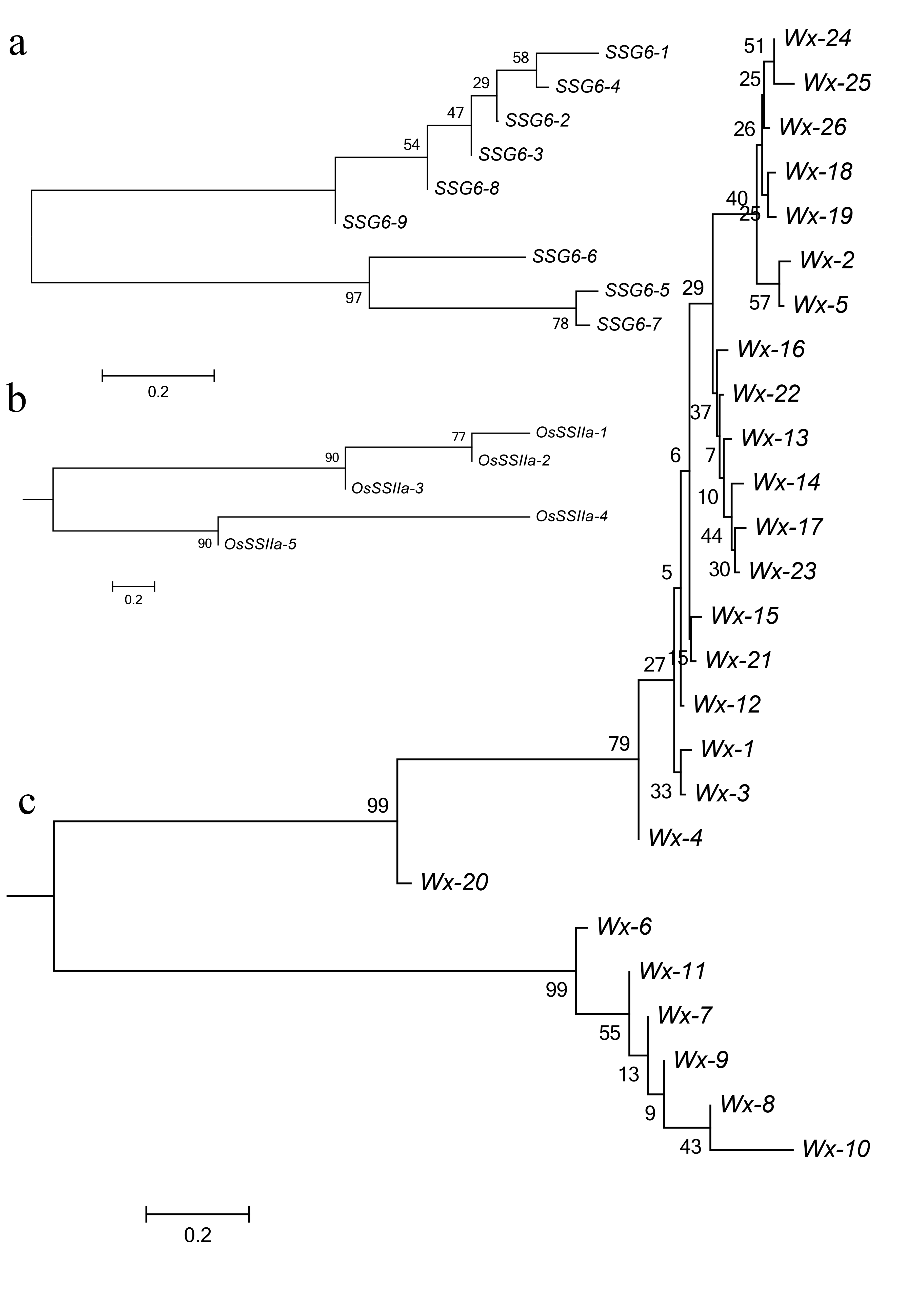


Fig.S3 Phylogenetic tree based on haplotypes of three keys genes in cultivated rice.
